# The Delaware Pain Database: a set of painful expressions and corresponding norming data

**DOI:** 10.1097/PR9.0000000000000853

**Published:** 2020-10-21

**Authors:** Peter Mende-Siedlecki, Jennie Qu-Lee, Jingrun Lin, Alexis Drain, Azaadeh Goharzad

**Affiliations:** a University of Delaware, Newark, Delaware, USA; b New York University, New York City, New York, USA

**Keywords:** Pain, Emotion, Facial expressions, Stimulus databases

## Abstract

**Introduction:**

Facial expressions of pain serve an essential social function by communicating suffering and soliciting aid. Accurate visual perception of painful expressions is critical because the misperception of pain signals can have serious clinical and social consequences. Therefore, it is essential that researchers have access to high-quality, diverse databases of painful expressions to better understand accuracy and bias in pain perception.

**Objectives:**

This article describes the development of a large-scale face stimulus database focusing on expressions of pain.

**Methods:**

We collected and normed a database of images of models posing painful facial expressions. We also characterized these stimuli in terms of the presence of a series of pain-relevant facial action units. In addition to our primary database of posed expressions, we provide a separate database of computer-rendered expressions of pain that may be applied to any neutral face photograph.

**Results:**

The resulting database comprises 229 unique (and now publicly available) painful expressions. To the best of our knowledge, there are no existing databases of this size, quality, or diversity in terms of race, gender, and expression intensity. We provide evidence for the reliability of expressions and evaluations of pain within these stimuli, as well as a full characterization of this set along dimensions relevant to pain such as perceived status, strength, and dominance. Moreover, our second database complements the primary set in terms of experimental control and precision.

**Conclusion:**

These stimuli will facilitate reproducible research in both experimental and clinical domains into the mechanisms supporting accuracy and bias in pain perception and care.

## Abstract 2

Supplemental Digital Content is Available in the Text.

We present a fully characterized face stimulus database focusing on expressions of pain, surpassing previous sets in terms of size, quality, diversity, and characterization.

## 1. Introduction

Facial expressions of pain—characterized by brow lowering, eyelid tightening, nose wrinkling, opening of the mouth, and raising of the upper lip and cheeks^32,44,69,79^—are social signals that communicate suffering and solicit aid.^13,14,41,79^ Painful expressions contain unique information distinct from other pain behaviors^11,28,47,66^ and are comparatively spontaneous and less subject to purposeful distortion vs self-report.^11^ Accordingly, painful expressions are given diagnostic weight over self-reports,^16^ even when judges know these expressions may be faked.^15,63^

Although pain has considerable impact on quality of life,^25,37,42,58^ lay individuals^65^ and clinicians are often too conservative in pain evaluation.^9,46,67^ These shortcomings are exacerbated by sociodemographic disparities in pain care: Black Americans are prescribed pain relievers less often and at lower doses,^8,23,34,55^ and the pain of Latinx Americans is also undertreated.^23,36,72^ Such gaps are mirrored in women^7,33,35^ and patients of low socioeconomic status.^39^ Addressing disparities in care requires understanding of their supporting psychological mechanisms. Unfortunately, this goal is constrained by stimuli that are limited in quantity, quality, and diversity.

### 1.1. Databases of painful expressions

Diverse, well-characterized stimulus sets are necessary to examine accuracy and bias in pain perception. However, most face databases do not include painful expressions.^45,51,75^ Instead, researchers present images or videos depicting body parts in painful scenarios^1,17,24,38^ or neutral/nonemotive faces experiencing pain.^10,81^ Other work pairs neutral faces with vignettes describing painful situations,^54,76^ which capture attributions of pain experience, rather than visual perception of pain.

However, some sets of painful expressions exist. The UNBC-McMaster set comprises videos of individuals experiencing shoulder pain during range-of-motion tests.^50^ Another set, the BP4D-Spontaneous database^83^ comprises a variety of spontaneously evoked expressions, including pain. Although these databases focus on experienced pain, smaller databases of posed painful expressions also exist.^70,73^ Together, these databases address the need for painful expression stimuli; however, several factors limit their usefulness.

### 1.2. Limitations of existing databases

Existing painful expression sets are relatively homogenous across race and gender. This lack of diversity poses an obstacle to understanding and eliminating disparities in pain care^19^ and stems from small sample sizes: Each set above comprises 50 or fewer subjects. The largest (BP4D-Spontaneous) contains only 20 white, 11 Asian, 6 black, and 4 Hispanic/Latinx subjects (23 female and 18 male^83^), making well-powered comparisons across race and gender difficult.

Existing pain stimulus databases also lack consistency. For example, in the UNBC-McMaster set, motion varies across subjects,^50^ occasionally obscuring their faces. Inconsistencies represent potential confounds in experimental designs, limiting the number of usable stimuli. Moreover, although dynamic stimuli enhance ecological validity, many designs require static images, and selecting video stills reduces quality.

Finally, although previous databases are validated on pain content, none provide details regarding social factors that influence pain judgments.^71,76,77,80^ Moreover, all stimuli should be comprehensively characterized in terms of social evaluations,^60^ racial prototypicality,^53^ and latent emotional content in neutral faces.^57,82^

### 1.3. The present work

Although painful expressions are a key social signal for communicating suffering, pain is underestimated in clinical settings and sociodemographic disparities in pain treatment are well-documented. However, research into the perceptual and psychological underpinnings of these disparities is limited by existing stimuli. We developed the Delaware Pain Database (DPD) to address these shortcomings with regards to size, homogeneity, characterization, and stimulus variability. To maximize scale, we used posed expressions of pain. Although spontaneous pain expressions may represent more ecologically valid stimuli,^15,63^ individuals are typically at chance in discriminating genuine vs posed pain expressions.^27,31,32,40,48,63^ Furthermore, similar action units (AUs) underlie both genuine and posed pain expressions.^16^

Our stimuli were extensively normed on pain-relevant dimensions (including attributions regarding strength, status, and dominance, non-pain emotional content, believability, and racial prototypicality) and were characterized on the presence of pain-relevant AUs, allowing researchers to make informed stimulus selections. All stimuli and corresponding norming information are available online (https://osf.io/2x8r5/).

## 2. Methods

### 2.1. Study 1: collecting and norming the Delaware Pain Database

#### 2.1.1. Collecting stimuli

We collected images of individuals posing multiple expressions of pain, at multiple intensities, in response to multiple prompts describing painful experiences.

##### 2.1.1.1. Models

Approximately 276 participants (“models”) were recruited and photographed at either the University of Delaware or New York University. Participants provided informed consent, in accordance with approval from institutional review boards at either university. Models were told that their images could be used in subsequent experiments, talks, or articles and might be manipulated visually or contextually (eg, paired with behaviors or labels indicating group membership). Models could opt out entirely or opt out of specific usages of their stimuli.

Eleven models were excluded from norming because of image issues (eg, blurry images, participants wearing glasses, or bangs covering forehead) or because they did not consent to experimental use of their images. Of the remaining 264 models, there are 29 female Asian, 25 male Asian, 36 female black, 33 male black, 20 female Hispanic/Latina, 17 male Hispanic/Latino, 44 male white, and 47 female white individuals (10 female and 3 male individuals self-identified as belonging to another racial group.) Self-reported age ranged from 18 to 34 years. Twenty-four models consented for their stimuli to be used in experiments, but not distributed online; their images are not posted online, but their ratings were analyzed.

##### 2.1.1.2. Stimuli collection

After providing informed consent, models completed a demographic survey. Next, models were seated in front of a plain white wall, 4 feet from a camera (Nikon Coolpix l330, Tokyo, Japan) on a tripod and lit by lamps. Models posed neutral facial expressions, followed by facial expressions representing how they would respond in a series of painful scenarios, at multiple intensities—specifically, levels 2, 5, and 8 of 10. Multiple images were taken for each prompt and level, and each session generated upwards of 50 images. Prompt and intensity level are recorded in the image filenames and norming datafile posted online. Ultimately, images taken at levels 2 and 5 were low in intensity, but intensity ranged considerably in level 8 images. Therefore, we determined it would be most feasible to proceed by limiting our database to (primarily) images posed at a level 8. For details, see Supplementary Materials (study 1, “Additional information regarding stimulus collection,” available at http://links.lww.com/PR9/A80).

##### 2.1.1.3. Formatting stimuli

**Figure 1.**
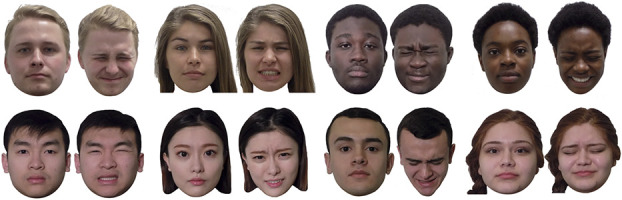
Sample stimuli of both neutral and pain expressions across race and gender within the Delaware Pain Database. All individuals depicted above gave permission for their likenesses to be used in published figures related to this database.

Neutral and painful stimuli were cropped to the head (from chin to top of hair), and backgrounds were removed (Adobe Photoshop CC, 2017). Each face was centered and straightened on a transparent 4 × 4-inch canvas (300 pixels/inch; Fig. 1). For details, see Supplementary Materials (available at http://links.lww.com/PR9/A80).

#### 2.1.2. Norming data collection

Previous research demonstrates the real-world behavioral consequences of social face evaluations,^60^ including racial phenotypicality.^53^ Moreover, perceived status,^76^ strength^77,80^ trustworthiness,^71^ and racial prototypicality^20^ specifically influence judgments of pain experience. Finally, latent emotional content in targets' faces^57,82^ could shape perception of subsequent expressions in dynamic stimuli.^22^ Therefore, we characterized both neutral and expressive stimuli on dimensions relevant to pain tolerance and experience.

##### 2.1.2.1. Neutral expressions

Six hundred sixteen paid MTurk participants (306 female; M_age_ = 35.12, SD_age_ = 10.84, 456 white/Anglo-American, 57 African American, 38 Asian, 39 Hispanic/Latinx, 8 Native American, and 18 identifying otherwise) rated a random subset of 285 formatted neutral-expression faces from the initial stimulus collection through Qualtrics. Each participant rated ∼27.23 (SD = 3.80) neutral faces, and each neutral face received ∼44.67 ratings (SD = 6.00). Two images were excluded from analyses because of blurriness. We did not select an a priori sample size for norming, but rather, assessed whether rating variability was appropriately small post hoc.

Participants rated each face on social dimensions (eg, attractiveness, trustworthiness, and status), resting emotional content (eg, sadness, disgust, and physical pain), and demographic features (perceived age, gender, race/ethnicity, and racial prototypicality). For details, see Supplementary Materials (study 1, “Additional information regarding stimulus norming,” available at http://links.lww.com/PR9/A80). Demographic vs nondemographic (social evaluations and emotion ratings) sections were blocked separately, with block order randomized across subjects. Within sections, question order was randomized.

For social and emotional judgments, perceived age, and racial prototypicality, we averaged across all ratings within a dimension for a given model. For demographic judgments, we calculated the proportion of raters who categorized a given model with a particular race or gender label. We also calculated modal race categorizations, based upon whichever race/ethnicity category received the most responses for a given model.

##### 2.1.2.2. Pain expressions

Although stimulus collection netted more than 3600 images, we pared this set down based upon quality (eg, too blurry), intensity (eg, posed at a level 2 or 5 intensity), variability (eg, essentially duplicate images within a model), and believability.

Thousand hundred fifty-eight paid MTurk participants (608 female; M_age_ = 35.71, SD_age_ = 11.06, 848 white/Anglo-American, 124 African American, 88 Asian, 61 Hispanic/Latinx, 9 Native American, 2 Pacific Islander, and 26 identifying otherwise) rated a randomized subset of 713 expressions through Qualtrics. On average, each participant rated ∼20.85 (SD = 6.15) emotional expressions, and each expression received ∼43.97 ratings (SD = 4.61). Three expressions were excluded from analyses because of blurriness. Note that these 713 images contain multiple versions of several stimuli that were edited to remove jewelry. We again assessed whether rating variability was appropriately small post hoc. For details, see Supplementary Materials (study 1, “Additional information regarding stimulus norming,” available at http://links.lww.com/PR9/A80).

Participants evaluated emotional expressions on the following dimensions: resemblance to specific emotions (eg, sadness, disgust, physical pain, etc) and believability/posedness of expression. For each expression, emotion was always rated before believability. Within each section, question order was randomized.

To calculate average ratings for each emotional content dimension, as well as perceived believability, we averaged across all ratings received within a dimension for a given expression. We also calculated pain specificity by determining the maximum value across responses for all emotions other than pain for a given expression and subtracting that non-pain maximum value from the average pain response for that expression.

##### 2.1.2.3. Objective ratings

Two groups of 2 independent raters measured the facial width-to-height ratio and median luminance of each neutral face in the database. As higher facial width-to-height ratio is associated with reduced attributions of pain experience^18^ and darker skin tones are associated with heightened thresholds for perceiving painful expressions independent of race,^20^ researchers may wish to control these factors (Supplementary Table 1, available at http://links.lww.com/PR9/A80).

##### 2.1.2.4. Reliability and variability

We estimated the reliability of all ratings of neutral and painful expressions. For brevity's sake, not all raters rated every image in our database and no image was rated by all raters, resulting in considerable missing data. Following the example of the Chicago Face Database (a large, diverse set of neutral faces^52^), we used an estimation of interdependence procedure^43^ to assess reliability. Once calculated, reliability estimates for each dimension were submitted to the Spearman–Brown Prophecy Formula.

Next, we assessed whether we had acquired enough ratings to arrive at an acceptably small margin-of-error for each dimension.^30^ Given that the 95% confidence interval surrounding a mean equals $1.96\times \frac{\sigma }{\sqrt{\text{n}}}$, this formula can be rearranged to solve for n, the sample size necessary for a desired margin-of-error, E:

$${\left(\frac{1.96\times \sigma }{\text{E}}\right)}^{2}.$$

We calculated the average SD across stimuli for each dimension. Using these values and the formula above, we assessed whether our samples of raters were appropriately large to afford us sufficiently small margins-of-error.

##### 2.1.2.5. Automatic and manual Facial Action Coding System coding

Given its scale, it was not feasible to manually code the entire DPD using the Facial Action Coding System (FACS;^21^). However, we used OpenFace (an open-source deep learning algorithm;^3,4^) to identify the presence/absence of pain-associated AUs. We also assessed OpenFace's reliability by contracting a trained FACS coder to manually code a subset (N = 100) of these stimuli.

We selected 50 pain-categorized and 50 non–pain-categorized images (balanced on race and gender) and subjected them to automated and manual coding. Next, we assessed the reliability (Cohen's kappa) of OpenFace's coding based on the trained FACS coder's judgments, focusing on pain-associated AUs,^32,44,69,79^ as well as the precision, recall, and decision accuracy of OpenFace's output. Furthermore, we compared the presence/absence of each AU in pain-categorized vs non–pain-categorized expressions within both manual and automatic coding. After determining which AUs were most reliable and pain-relevant, we calculated separate pain indices (summing the presence scores for AUs 4, 6, 7, 9, and 45; adapted from Ref. 69) from both the FACS coding and OpenFace output and assessed their relationship to pain intensity ratings collected in our initial norming. For details, see Supplementary Materials (study 1, “Additional information regarding FACS coding,” available at http://links.lww.com/PR9/A80).

### 2.2. Study 2: creating digitally rendered pain expressions

Although the diversity and variability of the DPD is a strength, some researchers may require additional control: One might wish to display identical expressions of pain across different targets. In study 2, we created and characterized a set of computer-generated painful expressions to facilitate this goal and provide a second set of stimuli—standardized pain expressions rendered on digital versions of DPD models.

#### 2.2.1. Initial expression creation

Creation and norming of a smaller set of computer-generated pain expressions was described in a recent investigation of racial bias in pain perception (experiments 6–7 in Ref. 56). That said, for details, see Supplementary Materials (study 2, “Additional information regarding expression creation,” available at http://links.lww.com/PR9/A80).

#### 2.2.2. Initial stimulus norming

##### 2.2.2.1. Participants

Eighty-one paid MTurk participants (45 female, M_age_ = 37.48, SD_age_ = 11.92; 52 white/Anglo-American, 8 African American, 11 Asian, 6 Hispanic, and 3 Native American) rated a randomized subset of 28 expressions drawn from a larger set of 41 through Qualtrics. Three expressions were mistakenly rated by all participants because of a randomization error. Aside from these expressions, each expression received ∼38.94 ratings on average (SD = 1.59); we used this value as a more conservative measure of ratings per stimulus for variability calculations. As in study 1, we assessed whether the variability of these ratings was appropriately small post hoc.

##### 2.2.2.2. Procedure

Expressions were rendered on the average face in FaceGen and were presented in color. Participants rated each expression on 8 emotions (sadness, disgust, surprise, threat, happiness, anger, fear, and physical pain) on 7-point Likert-type sliders (eg, “How much does this face look like it is in physical pain?”, 1 = not at all; 7 = extremely). Slider order was randomized within expression. Of the 28 randomly selected expressions presented to each participant, participants rated ∼21.21 (SD = 1.65) pain expressions, plus ∼6.79 (SD = 1.65) decoy expressions on average.

##### 2.2.2.3. Stimulus selection

First, we determined whether we had obtained enough ratings of our stimuli to minimize variability. Using the approach described in study 1,^30^ we calculated the number of raters needed to yield a margin-of-error within ± 0.5 units on each of our 7-point scales, 95% confidence level. Next, we analyzed the ratings of our 41 potentially painful expressions to identify those that were rated above the scale midpoint (4) for physical pain (pain intensity) and were rated as resembling pain more than any other emotion we collected ratings for (pain specificity; assessed through the paired *t* test).

## 3. Results

### 3.1. Study 1

#### 3.1.1. Reliability and variability

Overall, reliability was estimated to be high, ranging from 0.947 to 0.999 within neutral expressions and 0.983 to 0.997 within painful expressions (Table 1). However, as noted in the Chicago Face Database,^52^ given our large numbers of raters, these estimates may be inflated. Next, we determined we had recruited enough raters to achieve appropriately small margins-of-error surrounding our ratings (eg, ±0.5 on 7-point scales, 95% confidence level; see Supplementary Materials: study 1, “Variability analyses,” available at http://links.lww.com/PR9/A80).

**Table 1:** Reliability of subjective ratings of neutral and expression targets in the Delaware Pain Database.

Dimension	Neutral α	Pain α
Attractiveness	0.9917	—
Anger	0.9906	0.9917
Babyfacedness	0.9899	—
Believability	—	0.9849
Competence	0.9743	—
Disgust	0.9797	0.9906
Dominance	0.9890	—
Fear	0.9695	0.9877
Femininity	0.9985	—
Posedness	—	0.9829
Happiness	0.9944	0.9970
High status	0.9878	—
Intelligence	0.9797	—
Low status	0.9840	—
Masculinity	0.9983	—
Painfulness	0.9621	0.9957
Sadness	0.9897	0.9954
Strength	0.9960	—
Surprise	0.9471	0.9924
Threat	0.9822	0.9827
Trustworthiness	0.9882	—
Unusualness	0.9697	—

#### 3.1.2. Facial Action Coding System coding

Reliability, precision, recall, and decision accuracy of the initial OpenFace output are presented in Table 2, along with comparisons of each pain-associated AU in pain-categorized vs non–pain-categorized expressions within both manual and automatic coding. Based on these results, we concluded that the OpenFace coding was sufficiently reliable, and further, that AUs 4, 6, 7, 9, and 45 were the most reliable, pain-relevant AUs coded. Pain indices derived from these AUs were positively correlated with naive raters' judgments of pain intensity, both within the initial subset of expressions (manual coding: *r* = 0.746, *P* < 0.0001; OpenFace coding: *r* = 0.578, *P* < 0.0001) and across the full DPD (OpenFace coding: *r* = 0.484, *P* < 0.0001).

**Table 2:** Reliability, recall, precision, and accuracy of OpenFace automated coding, based on manual FACS coding of 50 pain-categorized and 50 non–pain-categorized images.

Action unit	Reliability (κ)	Recall	Precision	Accuracy	Presence in pain expressions (manual FACS)	Presence in nonpain expressions (manual FACS)	Presence in pain expressions (OpenFace)	Presence in nonpain expressions (OpenFace)
AU4*	0.451	0.812	0.958	0.810	0.98_a_	0.72_b_	0.94_a_	0.50_b_
AU6*	0.270	1.000	0.500	0.590	0.60_a_	0.20_b_	0.94_a_	0.68_b_
AU7*	0.357	0.934	0.845	0.800	0.86_a_	0.68_b_	0.94_a_	0.76_b_
AU9*	0.459	0.891	0.710	0.740	0.82_a_	0.28_b_	0.82_a_	0.56_b_
AU10	0.064	0.828	0.320	0.430	0.34_a_	0.24_a_	0.86_a_	0.66_b_
AU12	0.512	0.811	0.652	0.760	0.40_a_	0.36_a_	0.52_a_	0.40_a_
AU20	−0.005	0.400	0.098	0.570	0.14_a_	0.06_a_	0.42_a_	0.40_a_
AU25	0.899	0.945	1.000	0.950	0.56_a_	0.56_a_	0.56_a_	0.50_a_
AU26	0.485	0.763	0.552	0.800	0.12_b_	0.32_a_	0.18_b_	0.42_a_
AU45*	0.358	0.870	0.671	0.690	0.92_a_	0.16_b_	0.86_a_	0.56_b_
Average	0.385	0.825	0.631	0.714	0.574_a_	0.358_b_	0.704_a_	0.544_b_
Average in selected AUs:	0.379	0.901	0.737	0.726	0.836_a_	0.408_b_	0.900_a_	0.612_b_

Asterisks indicate AUs determined to be reliable and pain relevant based on these data. Reliability is measured in Cohen’s kappa values. Recall (eg, sensitivity) was calculated as the number of true positives divided by the sum of true positives and false negatives. Precision (eg, positive predictive value) was calculated as the number of true positives divided by the sum of true and false positives. The last four columns present the proportion of expressions demonstrating the presence of a given AU in pain-categorized and non–pain-categorized expressions, split by manual and automated coding. Values within a coding set with the different subscripts are significantly different from each other (*P* < 0.05; a > b).

AU, action unit; FACS, Facial Action Coding System.

#### 3.1.3. Correlational analyses

For brevity's sake, results of correlational analyses are presented in Tables 3–6. Overall, as expressions looked more fearful, disgusted, and sad, they tended to look more intensely painful, while happier, more surprised, threatening, and believable expressions tended to look less intensely painful. Moreover, cues to whiteness, masculinity, dominance, strength, threat, and pain gleaned from neutral faces were associated with increased pain intensity. Alternatively, cues to femininity, trustworthiness, attractiveness, intelligence, and happiness gleaned from neutral faces were all associated with decreased pain intensity. Moreover, expressions made by models categorized more frequently as South Asian, Pacific Islander, or Native American were rated as looking less intensely painful.

**Table 3:** Correlations between subjective ratings of pain and other emotional content in expression images.

	Fear (expression)	Anger (expression)	Disgust (expression)	Happiness (expression)	Sadness (expression)	Surprise (expression)	Threat (expression)	How believable? (expression)	How posed? (expression)
Pain (expression)	0.427†	0.039	0.211†	−0.458†	0.228†	−0.225†	−0.104†	−0.082*	0.054
Fear (expression)	—	0.007	0.173†	−0.445†	0.482†	0.212†	−0.046	0.153†	−0.141†
Anger (expression)	—	—	0.518†	−0.378†	−0.030	−0.126†	0.895†	0.079*	−0.049
Disgust (expression)	—	—	—	−0.512†	0.091*	−0.157†	0.386†	0.047	−0.043
Happiness (expression)	—	—	—	—	−0.448†	0.225†	−0.229†	0.038	0.087*
Sadness (expression)	—	—	—	—	—	−0.287†	−0.136†	0.412†	−0.435†
Surprise (expression)	—	—	—	—	—	—	−0.034	−0.117†	0.184†
Threat (expression)	—	—	—	—	—	—	—	0.031	0.013
How believable? (expression)	—	—	—	—	—	—	—	—	−0.913†

* *P* < 0.05.

† *P* < 0.001.

**Table 4:** Correlations between subjective ratings of pain and sociodemographic characteristtics of targets’ neutral images.

	Perceived age	%Male	%Female	%White	%Black	%Hispanic	%East Asian	%South Asian	%Pacific Islander	%Native American	%Other	Racial Prototypicality
Pain (expression)	0.092*	0.260†	−0.261†	0.130†	−0.031	−0.055	−0.045	−0.125†	−0.126†	−0.109†	−0.050	0.097*
Perceived age	—	0.231†	−0.227†	0.092*	0.012	−0.05	−0.124†	−0.057	−0.108†	−0.060	0.075	0.144†
%Male	—	—	−0.999†	0.105†	0.039	−0.100*	−0.078*	−0.106†	−0.162†	−0.191†	−0.060	0.171†
%Female	—	—	—	−0.105†	−0.039	0.100*	0.079*t	0.107†	0.163†	0.191†	0.051	−0.172†
%White	—	—	—	—	−0.586†	−0.237†	−0.372†	−0.455†	−0.380†	−0.172†	−0.174†	0.290†
%Black	—	—	—	—	—	−0.264†	−0.260†	−0.271†	−0.169†	−0.278†	−0.057	0.242†
%Hispanic	—	—	—	—	—	—	−0.111†	0.188†	0.379†	0.568†	0.107†	−0.670†
%East Asian	—	—	—	—	—	—	—	0.593†	0.274†	−0.003	−0.021	−0.118†
%South Asian	—	—	—	—	—	—	—	—	0.400†	0.259†	0.394†	−0.347†
%Pacific Islander	—	—	—	—	—	—	—	—	—	0.557†	0.059	−0.415†
%Native American	—	—	—	—	—	—	—	—	—	—	0.033	−0.432†
%Other	—	—	—	—	—	—	—	—	—	—	—	−0.333†

* *P* < 0.05.

† *P* < 0.001.

**Table 5:** Correlations between subjective ratings of pain and social evaluations of targets' neutral images.

	Masculinity (neutral)	Femininity (neutral)	Babyfacedness (neutral)	Trustworthiness (neutral)	Dominance (neutral)	Attractiveness (neutral)	Unusualness (neutral)	Strength (neutral)	High status (neutral)	Low status (neutral)	Competence (neutral)	Intelligence (neutral)
Pain (expression)	0.255†	−0.268†	−0.137†	−0.214†	0.121†	−0.197†	0.008	0.089*	−0.075	0.041	−0.164†	−0.167†
Masculinity (neutral)	—	−0.967†	−0.498†	−0.514†	0.686†	−0.528†	0.197†	0.635†	−0.275†	0.305†	−0.288†	−0.373†
Femininity (neutral)	—	—	0.456†	0.569†	−0.591†	0.629†	−0.214†	−0.524†	0.369†	−0.324†	0.381†	0.453†
Babyfacedness (neutral)	—	—	—	0.471†	−0.677†	0.293†	−0.025	−0.658†	0.198†	−0.195†	0.177†	0.265†
Trustworthiness (neutral)	—	—	—	—	−0.473†	0.726†	−0.329†	−0.346†	0.659†	−0.591†	0.790†	0.818†
Dominance (neutral)	—	—	—	—	—	−0.239†	0.117†	0.911†	−0.164†	0.334†	−0.158†	−0.280†
Attractiveness (neutral)	—	—	—	—	—	—	−0.422†	−0.168†	0.773†	−0.671†	0.754†	0.757†
Unusualness (neutral)	—	—	—	—	—	—	—	0.049	−0.273†	0.380†	−0.350†	−0.332†
Strength (neutral)	—	—	—	—	—	—	—	—	−0.144†	0.323†	−0.072	−0.215†
High status (neutral)	—	—	—	—	—	—	—	—	—	−0.807†	0.808†	0.833†
Low status (neutral)	—	—	—	—	—	—	—	—	—	—	−0.730†	−0.755†
Competence	—	—	—	—	—	—	—	—	—	—	—	0.929†

* *P* < 0.05.

† *P* < 0.001.

**Table 6:** Correlations between subjective ratings of pain and latent emotional content in targets' neutral images.

	Fear (neutral)	Anger (neutral)	Disgust (neutral)	Happiness (neutral)	Sadness (neutral)	Surprise (neutral)	Threat (neutral)	Pain (neutral)
Pain (expression)	0.076*	0.120†	0.085*	−0.078*	−0.017	−0.027	0.187†	0.117†
Fear (neutral)	—	0.149†	0.260†	−0.343†	0.683†	0.529†	0.085*	0.735†
Anger (neutral)	—	—	0.848†	−0.604†	0.261†	−0.171†	0.922†	0.429†
Disgust (neutral)	—	—	—	−0.544†	0.404†	−0.047	0.766†	0.570†
Happiness (neutral)	—	—	—	—	−0.574†	0.202†	−0.504†	−0.379†
Sadness (neutral)	—	—	—	—	—	0.092*	0.099*	0.659†
Surprise (neutral)	—	—	—	—	—	—	−0.124†	0.340†
Threat (neutral)	—	—	—	—	—	—	—	0.355†

* *P* < 0.05.

† *P* < 0.001.

#### 3.1.4. Demographics

Two hundred fifty-eight (37.9%) of all expressions received higher ratings of pain intensity vs other emotions. Almost twice as many painful expressions were obtained from male (versus female) models. Moreover, most painful expressions were obtained from black and white models. For a breakdown of pain-categorized expressions and neutral models by race and gender, see Table 7 (see also Supplementary Table 2, available at http://links.lww.com/PR9/A80).

**Table 7:** Race and gender breakdown of neutral and pain expressions available from models consenting to online distribution.

	Asian	Black	Latinx/Hispanic	White	Other	Total
A. Neutral expressions						
Male	23	29	15	44	2	113
Female	27	33	18	42	7	127
Total	50	62	33	86	9	240
B. Rated expressions						
Male	38	75	39	138	4	294
Female	65	82	49	89	22	307
Total	103	157	88	227	26	617
C. Available pain expressions						
Male	19	30	17	85	2	153
Female	14	23	10	26	3	76
Total	33	53	27	111	5	229

### 3.2. Study 2

#### 3.2.1. Variability

The largest SDs for evaluations of our computer-rendered expressions were observed for ratings of pain (avg. SD = 1.57). However, calculations suggested that our ratings were sufficiently stable given the number of raters we recruited (38.94 ratings per expression on average, vs 38.02 needed for a margin-of-error of ±0.5 units, 95% confidence level).

#### 3.2.2. Identifying painful expressions

Eleven of 41 expressions met criteria for pain intensity and specificity. Each expression was rated above the midpoint on painfulness (M = 5.18, all Ms > 4.65) and was rated as resembling pain more than any other emotion. The closest comparison was anger (average M = 2.41, Ms < 4.38, *P*s<0.0031).

Using FaceGen Modeller Pro, researchers can save and load expressions, rather than painstakingly producing them by hand. We recreated these 11 painful expressions using the Pro version and subjected them to a second norming survey (78 paid MTurk participants; 38 female, M_age_ = 33.16, SD = 9.38, 53 white/Anglo-American, 11 African American, 8 Asian, 5 Hispanic, and 1 Native American). Each expression was still rated above the midpoint on painfulness (average M = 4.85, Ms>4.13) and was rated as resembling pain more than any other emotion. The closest comparison was disgust (average M = 3.00, Ms < 3.41, *p*s<0.004). Moreover, an additional norming survey (45 paid Prolific participants; 23 female, M_age_ = 32.13, SD = 14.55, 23 white/Anglo-American, 6 African American, 4 Asian, 7 Hispanic, 1 Native American, and 4 identifying otherwise) determined that all but 1 expression was still robustly recognized as pain when rendered on a black target (average M = 4.32, Ms > 3.31; all other emotion Ms < 3.14 [disgust], all *P*s < 0.0031 [anger]). For details, see Supplementary Materials [study 2, “Additional information regarding stimulus norming.”] available at http://links.lww.com/PR9/A80).

**Figure 2.**
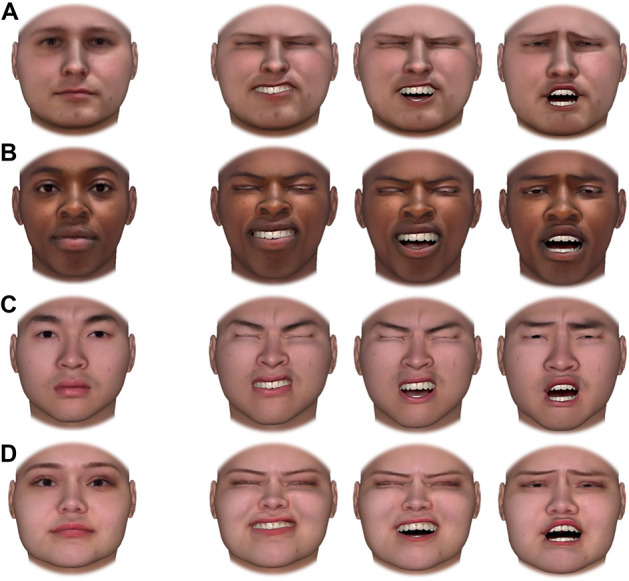
Sample of pain expressions meeting inclusion criteria in Study 2 rendered onto stimuli imported into FaceGen. The column of faces to the far left represents each targets' neutral expression, while the 3 columns at the right represent each target with the same 3 expressions of pain. White male (row A), black female (row B), Asian male (row C), and Latina female (row D) are the digitally rendered versions of targets from Figure 1 (since FaceGen stimuli are rendered without hair, these faces have been vignetted to remove cues to “baldness.”). All individuals depicted above gave permission for their likenesses to be used in published figures related to this database.

Materials posted online can be used to recreate all expressions detailed above. Researchers can present multiple targets, potentially varying in race and/or gender, making objectively equated expressions of pain. To demonstrate the utility of this approach, we created such stimuli for a small subset of DPD models and vignetted them to remove “baldness” cues (Fig. 2). These stimuli are also available online.

## 4. Discussion

Facial expressions communicating the presence of pain and severity of suffering represent a vital social signal.^11,13,14,79^ However, although facial expressions of pain are processed rapidly and spontaneously,^12,64,74,78^ generalize across cultures,^6^ and drive empathic responses,^5,26,29^ pain is underestimated in clinical settings^68^ and sociodemographic disparities in pain care persist.^2,23^

Understanding gaps in pain care requires large, diverse sets of high-quality stimuli. Although several databases of painful expressions exist,^50,70,72,83^ each has substantial drawbacks: lack of racial and gender diversity, consistency in quality and formatting, and overall volume. Therefore, we developed the DPD, a large-scale face database focusing on expressions of pain. In addition to its scale and diversity, the DPD was characterized across numerous social, emotional, and demographic dimensions relevant to pain. Several factors (resemblance to negative emotions such as anger and sadness, cues to whiteness and masculinity) were positively correlated with higher subjective ratings of pain intensity.

The variability of the DPD provides significant advantages in ecological validity and represents a major strength of this database. However, some researchers may wish to equate expressions across targets. Consequently, we provide additional stimuli using models from the DPD, upon which normed pain expressions have been digitally rendered. Using identical expressions across targets provides greater precision and internal validity. The necessary materials are available online, so that researchers may apply these painful expressions to any desired neutral face using FaceGen.

Similarly, norming data for both stimulus sets is available, allowing researchers to make informed selections. Given recent focus on scientific replicability and reproducibility,^59,61,62^ researchers must be open and transparent about their stimuli. Is an effect observed across a wide range of stimuli, or just a subset that a researcher continually uses? Did a researcher select stimuli based upon principled criteria or convenience? Experiments using the DPD stimuli will be more easily reproduced and more directly comparable, abating replication issues arising from inconsistencies across experiments using uncharacterized sets.

### 4.1. Potential applications

The stimuli of the DPD will have many fruitful applications. For example, psychologists or clinicians studying judgments of pain experience will benefit from these stimuli. Moreover, researchers studying the neural mechanisms supporting empathy for pain or the visual perception of pain should be well-served by this resource. In addition, this stimulus database will support new research on pain care disparities. Although existing stimulus sets lack racial diversity or focus on specific racial comparisons, the DPD allows researchers to examine whether biases in pain perception and treatment generalize across multiple racial categories. The gender diversity of our database will also allow researchers to examine the effects of gender on pain-related processes and to take a more intersectional approach to studying disparities in pain care.

Because of this diversity, researchers may better examine how pain-related outcomes vary based on these sociodemographic variables, promoting better understanding of disparities in pain care.^19^ Indeed, stimuli from the DPD have been used to demonstrate that racial bias in pain perception facilitates disparities in treatment in white perceivers,^56^ and further, that racial bias in pain perception is exacerbated by bottom-up and top-down cues to racial prototypicality.^20^

### 4.2. Limitations and conclusion

Although the DPD offers improved utility and flexibility, some potential limitations remain. First, models were not photographed experiencing pain, and therefore, their posed expressions are not truly “genuine.” Although this concern is valid, perceivers generally perform at chance in distinguishing between real and posed pain.^48,49,63^ Moreover, for researchers apprehensive about using posed stimuli, we provide subjective ratings of believability and “posedness.” Researchers may select stimuli surpassing a desired threshold for believability and balance stimuli across groups accordingly.

In addition, the DPD stimuli are static. Dynamic expressions, like those in the BP4D-Spontaneous set,^83^ the UNBC-McMaster set,^50^ or those derived in recent cross-cultural investigations of painful expressions,^6^ allow researchers to examine the temporal dynamics of pain perception with precision. One may circumvent this limitation by morphing neutral and painful images from the DPD to create dynamic expressions.^20,56^ Using morphing software, a researcher can produce morphs representing points along the continuum from one face to another or generate a video transitioning from a neutral face to a painful face.

Furthermore, white models and raters are both disproportionately represented in the database. Although correspondence in pain intensity ratings was high across rater race, there was some evidence of small in-group biases in pain ratings (Supplementary Materials: study 1, “Assessing in-group bias in pain ratings,” available at http://links.lww.com/PR9/A80). Raters rated pain expressions made by own-race models higher on pain intensity, and this effect was statistically significant within white raters. Although this in-group bias is diluted across the full set of raters, pain may be somewhat overestimated on white models' faces. However, the consequence of this bias is that comparisons between white and nonwhite models equated on pain ratings will be more conservative tests of racial bias in pain perception, since pain would be comparatively underestimated on nonwhite models' faces.

Finally, stimuli in the DPD were all between 18 and 34 years old. We will continue to expand the database and recruit participants above and below this age range. In addition, we will continually add models from historically understudied populations, including Latinx, Middle Eastern, and Asian individuals. Consequently, our online repository of images and norming data will be continually updated and maintained. Moving forward, the DPD will serve as a useful tool for researchers studying pain at multiple levels of analysis—from perceptual processes involved in the visual recognition of pain, to clinical outcomes associated with disparities in pain treatment.

## Disclosures

The authors have no conflicts of interest to declare.

## Appendix A. Supplemental digital content

Supplemental digital content associated with this article can be found online at http://links.lww.com/PR9/A80.

## Supplementary Material

SUPPLEMENTARY MATERIAL
