# Supplementary material for: The Delaware Pain Database: a set of painful expressions and corresponding norming data
Source: Pain Rep. 2020 Oct 21;5(6):e853. doi: 10.1097/PR9.0000000000000853 (PMC7587421; doi:10.1097/PR9.0000000000000853)
Supplement: SUPPLEMENTARY MATERIAL [file painreports-5-e853-s001.docx]

**Supplementary Tables**

|  | Facial width-to-height ratio (neutral) | Median facial luminance (neutral, face only) | Median facial luminance (neutral, full stimulus) | Mouth width-to-height ratio (expression) | Left eye width-to-height ratio (expression) | Right eye width-to-height ratio (expression) |
| --- | --- | --- | --- | --- | --- | --- |
| Pain (expression) | -.010 | .013 | .098* | .131** | .495** | .512** |
| Facial width-to-height ratio (neutral) | - | .151** | -.126** | .041 | -.038 | -.009 |
| Median facial luminance (neutral, face only) | - | - | .458** | .047 | -.036 | -.030 |
| Median facial luminance (neutral, full stimulus) | - | - | - | .074 | -.026 | -.035 |
| Mouth width-to-height ratio (expression) | - | - | - | - | .175** | .170** |
| Left eye width-to-height ratio (expression) | - | - | - | - | - | .822** |

**Supplementary Table 1.** Correlations between subjective ratings of pain and neutral images.

**p* < .05, ***p* < .001

| A. Neutral expressions |  | Asian | Black | Latinx/  Hispanic | White | Other | Total |
| --- | --- | --- | --- | --- | --- | --- | --- |
|  | Male | 25 | 33 | 17 | 44 | 3 | 122 |
|  | Female | 29 | 36 | 20 | 47 | 10 | 142 |
|  | Total | 54 | 69 | 37 | 91 | 13 | 264 |
|  |  |  |  |  |  |  |  |
| B. Rated expressions |  | Asian | Black | Latinx/  Hispanic | White | Other | Total |
|  | Male | 41 | 91 | 40 | 138 | 7 | 317 |
|  | Female | 72 | 86 | 54 | 98 | 33 | 343 |
|  | Total | 113 | 177 | 94 | 236 | 40 | 660 |
|  |  |  |  |  |  |  |  |
| C. Available pain expressions |  | Asian | Black | Latinx/  Hispanic | White | Other | Total |
|  | Male | 21 | 39 | 17 | 85 | 5 | 167 |
|  | Female | 14 | 26 | 12 | 29 | 10 | 91 |
|  | Total | 35 | 65 | 29 | 114 | 15 | 258 |

**Supplementary Table 2.** Race and gender breakdown of neutral images and pain expressions, *including* models *not* consenting to online distribution.

**Study 1: Supplementary Materials**

**Additional information regarding stimulus collection**

**Model recruitment.**

Recruitment for this project took place in three waves across two institutions. The initial phase of recruitment was entirely a sample of convenience: 29 male individuals (specifically, individuals who were acquaintances of the first author and who had previous acting experience) were recruited on a volunteer basis and photographed at New York University. Subsequently, 181 additional male and female individuals (“models,” throughout) were recruited and photographed from several PSYC100 courses at the University of Delaware in exchange for research credits required for course completion. After this initial wave, to obtain a maximally diverse pool of stimuli, we used a combination of convenience and snowball sampling to recruit an additional 66 models. 18 received research credit for participation, while the other 48 were compensated at the end of a 30-minute session with $5.

**Multiple versions of stimuli.** For a small subset of models, multiple formatted versions are available. The original formatted set for which norming data was collected was cropped to a square canvas, which meant that some models’ hair was cut off in some images. In these cases, we re-formatted these images—either so their hair could extend beyond the square canvas or so that their hair could be cropped in a more naturalistic fashion. In addition, two models from the first round of stimulus collection (1 Black male, 1 White male) posed for stimuli twice, in two separate sessions.

Finally, eight male models (7 Black male, 1 Asian male) were mistakenly photographed wearing ear piercings, while 12 models (5 Black female, 3 White female, 1 Asian female, 2 Hispanic/Latina female, 1 biracial female) were mistakenly photographed wearing nose rings. Edited versions of these models’ stimuli (e.g., earrings/nose rings removed via Photoshop) were created and normed, as were the originals. These edited stimuli are not counted as separate models in the tallies in the main text. Analyses pertaining to norming data includes the edited versions of these stimuli, not the original versions.

**Differences between stimulus collection waves.** The NYU and UD stimulus collection waves differed in a few ways. First, photographs at NYU were taken with an iPhone 5S. Second, models at NYU did not hold a whiteboard during their photo sessions. As a result, while the order in which prompts were given and images were collected was always the same, information on which specific pain experience was being modeled in each image was not preserved. Third, models at NYU posed expressions in response to two additional prompts: having one’s arm submerged in a bucket of ice water for a prolonged period of time and having lemon juice applied to a paper cut on the webbing between one’s fingers. Finally, models at NYU did not pose subsequent additional emotional expressions (e.g., happiness, fear, etc.) and several were photographed outside of the running room context.

**Instructions.** First, models were instructed to make a neutral facial expression. Subsequently, models were asked to pose expressions in response to five specific prompts describing potentially painful experiences: receiving an electric shock via electrode, receiving burning heat pain via thermode, cutting one’s index finger while chopping garlic, experiencing a migraine while at work, and experiencing internal pain localized to the abdomen. We requested that models portray how they would respond in each scenario, that they localize their responses to their faces, and that they were not to obscure any part of their face while posing their response (e.g., by covering their face with their hands, or by turning away from the camera).

For each prompt, models were instructed to pose expressions at three levels of pain – a 2 (“annoying, but you can almost ignore it”), 5 (“definitely painful, but you can grit^[[1]](#footnote-1)^ your teeth through it”), and 8 (“almost unbearable, the most pain you’d be willing to experience”) on a scale from 1 to 10. After completing the pain expressions, models also posed a variety of other emotional expressions (i.e., happiness, sadness, fear, anger, and surprise) that were not included in the norming tasks. Researchers always took multiple images for every prompt. In order to keep track of which image corresponded to which prompt, models were asked to hold a small whiteboard, which the research assistant conducting the session marked each image’s number upon (e.g., 1 = neutral expression, 2a = electric shock at level 2, 2b = electric shock at level 5, etc.).

When models did not intensify their expressions from level to level, they were directed to do so. To enhance variability within the stimulus set, models who made similar expressions across prompts were encouraged to try out different facial configurations (e.g., eyes open vs. closed, mouth closed vs. teeth gritted). When models didn’t change or intensify their expressions from level to level, the author or research assistant who was conducting the photo session offered a suggestion such as “I didn’t see much change in your expression that time… remember, this is a(n) [LEVEL] out of 10 pain.” If this statement didn’t elicit enough of a change, we gave a more direct request, such as, “Can you go a little bigger than that?” As for variability, if it appeared that a model was essentially making the same expression across all prompts, we would offer a suggestion like, “Feel free to change things up if you feel like you’d make different types of expressions for different situations.” If this statement didn’t prompt them to vary their expression, we would give a more direct, such as, “Can you try something a little different?”

Given the large number of images obtained for each model (~50), we accrued approximately 3,600 images in total. As it would have not been feasible to rate all images that were collected, we pared this sum down to approximately 700. Many of the excess images were taken at levels 2 and 5, which, as we describe in the main text, went unrated in all but a few cases (N = 13). (In those cases, the level 8 expressions were unusable due to quality, but we normed a level 5 expression in the hopes of not excluding a model completely.) The additional excess was almost entirely due to the fact that researchers/research assistants were taking photos in continuous shooting mode during the photo sessions. As such, the rest of the unrated images were typically stimuli that were lacking in quality (e.g., they were blurry because the model was moving either their face or body at the moment the photo was taken) or they were essentially duplicates of other images selected for inclusion. Further, since models posed one pain expression per prompt, RAs selecting stimuli for editing and rating were essentially looking for the frame that best captured the model’s pose.

Some additional images were excluded from norming due to obvious believability concerns. Posing painful expressions for a stranger holding a camera can be an awkward prospect and—especially early in the sessions—it was not uncommon for models to laugh or be somewhat half-hearted in their expressions. As a result, we often had to remove images that simply wouldn’t have been suitable for use in research. While we did also obtain believability ratings of the stimuli selected for norming and could conceivably have triaged stimuli based on an initial believability survey, these excluded stimuli typically failed to capture pain so obviously that it would have been wasteful to have them rated.

**Additional information regarding stimulus norming**

**Neutral ratings.**

***Participants.*** Of the 616 Mechanical Turk participants who completed the neutral expression norming, there were 306 women, 302 men, and 8 individuals who self-identified with another gender category. Moreover, 456 of these individuals identified as White/Caucasian, 57 identified as African American, 38 identified as Asian, 39 identified as Hispanic/Latinx, 8 identified as Native American, and 18 identified as belonging to another racial group.

***Ratings.*** Here we offer more specific details on what dimensions participants were asked to rate neutral expressions on. Participants evaluated each face on a series of social dimensions (attractiveness, dominance, masculinity, femininity, intelligence, high status, low status, unusualness, strength, babyfacedness, competence, trustworthiness; each rated on a 7-point Likert-type scale: e.g., “How attractive does this face look?”, 1 = not at all, 7 = extremely), and also rated each face in terms of its resting emotional content (sadness, disgust, surprise, threat, happiness, anger, fear, and physical pain; each rated on a 7-point Likert-type scale: e.g., “How sad does this face look?”, 1 = not at all, 7 = extremely).

In separate blocks, participants rated each face in terms of its demographic features (perceived age, 0-100 years old; gender, “Male,” “Female,” or “Other” with free response; race/ethnicity, “White,” “African American,” “Hispanic,” “East Asian,” “South Asian,” “Native American,” “Pacific Islander,” or “Other” with free response; and racial prototypicality, in response to the question “How typically [categorized race] does this person look?” from 1, “Less typically [categorized race] looking,” to 5, “Very typically [categorized race] looking,” where [categorized race] represents piped responses from the race/ethnicity question, excluding “Other” responses). All responses were made within an unlimited response window.

Average ratings of neutral images are collected in columns E through AM of the first tab in the file “DelawarePainDatabase_StimulusCharacterization_forOSF.xlsx” posted in the “Norming Data” folder on OSF (<https://osf.io/2x8r5/>).

**Pain ratings.**

***Participants.*** Of the 1158 Mechanical Turk participants who completed the pain expression norming, there were 608 women, 541 men, 2 transgender individuals, and 7 individuals who self-identified with another gender category. Moreover, 848 of these individuals identified as White/Caucasian, 124 identified as African American, 88 identified as Asian, 61 identified as Hispanic/Latinx, 9 identified as Native American, 2 identified as Pacific Islander, 26 identified as belonging to another racial group.

***Ratings.*** Here we offer more specific details on what dimensions participants were asked to rate emotional expressions on. Participants made a series of evaluations of each emotional expression along the following dimensions: resemblance to specific emotions (sadness, disgust, surprise, threat, happiness, anger, fear, and physical pain on a 7-point Likert-type scale: e.g., “How sad does this face look?”, 1 = not at all; 7 = extremely) and believability of expression (9-point Likert-type scale: “How believable is this expression?”, 1 = not at all believable, 9 = very believable; “How posed does this expression look?”, 1 = not at all posed, 9 = very posed). For each expression, emotion ratings were always presented prior to believability ratings. Within each section, the order of individual questions (e.g., “How angry does this face look?”) was randomized. All responses were made within an unlimited response window.

Average ratings of expression images are collected in columns AN through BA of the first tab in the file “DelawarePainDatabase_StimulusCharacterization_forOSF.xlsx” posted in the “Norming Data” folder on OSF (<https://osf.io/2x8r5/>).

**Inclusion of decoy stimuli.** In several early waves of norming data collection, 120 decoy expressions of other emotions (happiness, sadness, fear, surprise) were randomly intermixed with the painful expressions (3.44 decoys per subject). These decoys were included to broaden the range of expressions that participants saw, in order to ensure that their ratings were relative to a variety of emotional displays. That being said, given the number of expressions that were not perceived as painful by raters, we ultimately concluded that this was less of a pressing concern. As such, latter waves that collected data on edited version of stimuli (e.g., earrings or nose rings digitally removed) did not include decoy expressions.

**Objective ratings.** Median luminance was collected two ways. First, raters assessed the median luminance of each face (excluding ears, necks, hair, and background). Second, raters assessed the median luminance across the entire image (e.g., cropped head on transparent background). For expression images, these raters reported the type of expression (four different combinations of eyes-close/open and mouth-close/open), and calculated the ratio between the width of mouth from corner to corner and the height from upper lip topline to bottom lip bottom line (mWHR), the ratio between the width of left eye from corner to corner and the height from upper eyelid to lower eyelid (leWHR), the ratio between the width of right eye from corner to corner and the height from upper eyelid to lower eyelid were collected (reWHR) of each expression.

Prior to rating those features, four research assistants were trained using the same materials. Two research assistants independently measured the first two dimensions of all neutral faces using a ruler and Adobe Photoshop software. The other two research assistants completed their measurements of the last four dimensions on all pain faces using the same standard ruler. Reliability across objective measurements was very high (median luminance values = .993 for the face-only images, while these ratings were equal across raters for the full stimuli calculations, fWHR: .968, mWHR: .980; leWHR: .927; reWHR: .938), as was correspondence between expression categorizations (agreement on 94.8% of expressions). Once both groups of raters completed their measurements, any discrepancy in expression categorizations of the same pain face was discussed and resolved. When two raters differed by more than one standard deviation above the average inter-rater difference for a given objective dimension, a third rater was brought in to evaluate the stimulus in question on that dimension. The third rater’s measurement then replaced whichever of the first two raters was further from the third. Average values were then computed across raters.

Objective ratings are collected in columns BB through BG of the first tab in the file “DelawarePainDatabase_StimulusCharacterization_forOSF.xlsx” posted in the “Norming Data” folder on OSF (<https://osf.io/2x8r5/>).

**Additional information regarding FACS coding.** As discussed in the main text, we could not feasibly code the entire DPD manually using the Facial Action Coding System (FACS; [4]). However, upon consultation with an expert in both automatic and manual FACS coding, we devised a strategy that would allow us to determine the reliability of an automated coding approach (OpenFace; an open-source deep learning algorithm that can be used to evaluate faces; [1,2]) and then, if satisfied, to submit the entire DPD for automated FACS coding.

First, we selected 50 pain categorized and 50 non-pain categorized images from the DPD that were balanced in terms of race and gender (e.g., equal numbers of Black, White, Latinx, Asian, and other race targets in each category and equal numbers of male and female targets in each category). Second, these images were coded by OpenFace on the presence/absence of activation of 18 action units: 1, 2, 4, 5, 6, 7, 9, 10, 12, 14, 15, 17, 20, 23, 25, 26, 28, and 45. Subsequently, a trained and certified FACS coder coded this same set of images on presence/absence of these same 18 action units. Third, we assessed reliability between the scoring of our trained FACS coder and OpenFace’s output within pain-relevant action units. In light of previous work [3,5,7], we focused on AU4 (brow lowerer), AU6 (cheek raiser), AU7 (lid tightener), AU9 (nose wrinkler), AU10 (upper lip raiser), and 20 (lip stretcher) as our primary pain-relevant action units. While AU43 (eyes closed) was not coded by OpenFace, we also examined AU45 (blink) as a substitute. We also assessed AU12 (lip corner puller), AU25 (lips part), and AU26 (jaw drop). We calculated reliability (Cohen’s kappa) between our manual FACS coding and the OpenFace output, as well as the precision (e.g., positive predictive value; (true positives) / (true positives + false positives), recall (e.g., sensitivity; (true positives) / (true positives + false negatives), and decision accuracy of the OpenFace output, based on the manual coding. In addition, we compared the proportion of stimuli that activated each AU in pain-categorized versus non-pain-categorized expressions within both manual and automatic coding.

We then integrated across these measures in order to determine a) whether the OpenFace coding was sufficiently reliable and b) if so, which AUs were most reliable and pain-relevant. There was a strong correspondence between the OpenFace output and the manual FACS coding, reliability was low in AU10 and AU20. Moreover, AUs 12, 25, and 26 were not significantly more likely to be present in pain-categorized versus non-pain-categorized expressions. As such, we determined that AUs 4, 6, 7, 9, and 45 were most reliable and pain-relevant.

We calculated a pain intensity index across these five AUs—a simple sum of their presence/absence values for each expression. Ultimately, this index is essentially a simplified version of the Prkachin & Solomon Pain Index ([7]; AU4 + max(AU6,AU7) + max(AU9,AU10) + AU43), with AU10 removed and AU43 swapped for AU45. We calculated this index separately based on the automated and manual coding and assessed the relationship between this index and our naïve raters’ judgments of pain intensity. As demonstrated in the main text, this relationship was strong and positive in both cases (FACS coded: r = .746, p < .0001; OpenFace coded: r = .578, p < .0001). Satisfied that OpenFace produced a reliable characterization of our test images, we subjected the rest of the database to automated coding. A comparison of the presence/absence of these five pain-relevant AUs across the full set of pain-categorized versus non-pain-categorized images can be found below.

These data are collected in columns BQ through CJ of the first tab in the file “DelawarePainDatabase_StimulusCharacterization_forOSF.xlsx” posted in the “Norming Data” folder on OSF (<https://osf.io/2x8r5/>). A comparison between the manual FACS coding (columns D-V) and automated OpenFace coding (columns Z-AR) of the selected 100 expressions can be found in the last tab of the same sheet.

**Assessing in-group bias in pain ratings.** Raters in the neutral and pain expression norming samples described in the main text were disproportionately White (74.0% White in the neutral norming survey, 73.2% in the pain norming survey). As such, if perceivers display an in-group bias in rating pain intensity, it’s possible that the ratings we obtained reflect systematic overestimations of pain portrayed by White models. To address this, we compared ratings of Black, Asian, Hispanic, and White models between Black, Asian, Hispanic, and White raters, based on a reviewer’s suggestion. Given the comparatively small number of models belonging to the “other race” category and the comparatively small number of raters identifying as Native American, Pacific Islander, or another racial group, we did not include raters or models identifying with these groups in this analysis. Since such an in-group bias would be primarily problematic if it shaped judgments of pain intensity within the expression images, we limited our additional analyses to ratings on this dimension within those images.

Specifically, we a) compared overall correlations of pain intensity ratings across all expressions between these four groups of raters (e.g., are White raters’ ratings of pain intensity across the full set of models related to Asian raters’ ratings of those models?), b) compared correlations between pain intensity ratings within each set of models by each set of raters across all expressions (e.g., are Black raters’ ratings of Black models’ pain intensity related to White raters’ ratings of those specific models?), c) compared differences between pain intensity ratings of the pain-categorized expressions of each set of models made by each set of raters (e.g., via a mixed-factorial ANOVA, we assessed whether ratings of pain vary as a function of model race, rater race, and the interaction of the two), and d) assessed specifically whether perceivers rated pain portrayed by in-group models differently than pain portrayed by out-group models (via paired t-tests).

**Inclusion of stimuli in analyses.** The analyses in the main text (as well as this supplement) *do* include ratings of individuals who did not consent for their stimuli to be made available for use online, as well as *both* sets for those models whose photos were taken in multiple sessions. However, only ratings of retouched images (to remove jewelry) were used for those affected models.

**Additional results**

**Variability analyses.** Instead of conducting power analyses prior to data collection for the norming samples, we chose to assess whether we’d acquired enough raters to arrive at an acceptably small margin-of-error for each dimension *post hoc* [28]. As described in the main text, the 95% confidence interval surrounding a mean equals $1.96 \times\frac{\sigma}{\surd n}$ , and therefore, this formula can be rearranged to solve for *n*, the sample size necessary to obtain a prescribed margin of error, *E*:

$$\left( \frac{1.96 \times\sigma}{E} \right)^{2}$$

After norming data collection was complete, we calculated the average standard deviation across stimuli within each specific set of ratings. Using these values and the formula above, we calculated the number of raters needed to yield a margin-of-error *E* within +/- .5 units on each of our 7-point scales, with 95% confidence.

The largest standard deviations for subjective social and emotional evaluations of neutral faces were observed for ratings of babyfacedness (avg. *SD* = 1.64) and resting sadness (avg. *SD* = 1.52), respectively. That being said, our calculations suggested that our ratings were sufficiently stable given the number of neutral stimulus raters we recruited (44.67 ratings per neutral face on average, versus 41.15 and 35.70 recommended, respectively). Similarly, the largest standard deviations for evaluations of emotional expressions were observed for ratings of disgust (avg. *SD* = 1.65), suggesting that we’d also recruited a sufficient number of emotional expression stimulus raters (43.97 ratings per expression on average, versus 41.77 recommended).

We made similar calculations for additional ratings that were *not* made on 7-point scales – specifically, age (rated on a scale from 0 to 100 years), racial prototypicality (rated on a scale from 1 to 5), and believability (rated on a scale from 1 to 9). Using the same approach, we determined that given the observed average standard deviation across stimuli for each of these dimensions suggested that we’d obtained a sufficient number of ratings to yield a +/-2-year margin-of-error for age ratings (35.80 ratings recommended), +/-.3 units for racial prototypicality ratings (37.94 ratings recommended), and +/-.75 units for judgments of how posed the expressions look (39.36 ratings recommended), with 95% confidence.

**Correlations with objective measurements.** Beyond the correlations presented in the main text, we also examined whether any objective characteristics of the images in our set influenced perceptions of pain intensity. Specifically, we tested the influence of fWHR, median luminance, and a series of measures of the eyes and mouth (e.g., mWHR, leWHR, and reWHR). While neither the fWHR (*r* = -.010, *p* = .793) nor face-only luminance (*r* = .013, *p* = .742) of neutral images was associated with evaluations of pain intensity in emotional expressions, mWHR (*r* = .131, *p* = .001), leWHR (*r* = .495, *p* < .001), and reWHR (*r* = .512, *p* < .001) were each positively associated with evaluations of pain intensity, suggesting that expressions portraying tightly shut mouths or eyes were rated as looking more intensely painful. Interestingly, full-stimulus luminance was also positively associated with evaluations of pain intensity (*r* = .098, *p* = .012), suggesting that there might be some information outside the facial features (e.g., hair, etc.) that might be associated with these judgments. (See Supplementary Table 1.)

**Effects of expression type and prompt on subjective ratings.** Within the 258 pain-categorized expressions, we examined how pain intensity, specificity, and believability varied as a function of the type of expression models’ posed – specifically, whether they closed or opened their eyes and closed or opened their mouths. We observed that subjective ratings of pain intensity varied significantly between the four expression types (*F*(3,254) = 6.36, *p* < .001, η_p_^2^ = .07). Eyes-closed/mouth-open expressions (*M* = 4.30, *SD* = 0.79, *N* = 107) were rated as being more intense than eyes-open/mouth-closed (*M* = 3.52, *SD* = 0.52, *N* = 8; *p* = .005) or eyes-open/mouth-open expressions (*M* = 3.67, *SD* = 0.68, *N* = 23; *p* < .001). In turn, eyes-closed/mouth-closed expressions (*M* = 4.18, *SD* = 0.74, *N* = 120) were also rated as being more intense than eyes-open/mouth-open expressions (*p* = .004) and eyes-open/mouth-closed expressions (*p* = .017).

Pain specificity—calculated as the subjective rating of pain intensity minus the value of whichever other emotion received the highest intensity rating—also varied significantly between the four expression types (*F*(3,254) = 9.10, *p* < .001, η_p_^2^ = .10), following a very similar pattern as pain intensity. Eyes-closed/mouth-open expressions (*M* = 1.39, *SD* = 0.79, *N* = 107) were rated as more pain-specific than eyes-open/mouth-closed (*M* = 0.47, *SD* = 0.49, *N* = 8; *p* = .001) or eyes-open/mouth-open expressions (*M* = 0.66, *SD* = 0.55, *N* = 23; *p* < .001). In turn, eyes-closed/mouth-closed expressions (*M* = 1.26, *SD* = 0.74, *N* = 120) were also rated as more pain-specific than eyes-open/mouth-open expressions (*p* < .001), as well as eyes-open/mouth-closed expressions (*p* = .004). Subjective ratings of how believable (*F*(3,254) = 0.77, *p* = .513, η_p_^2^ = .01) or how posed (*F*(3,254) = 0.92, *p* = .431, η_p_^2^ = .01) expressions looked did not vary as a function of expression type.

Finally, we measured how these same measures varied as a function of the prompt that targets were responding to. (Prompt data was not recorded or recoverable for targets who were photographed at NYU. As a result, data for those subjects’ pain-categorized expressions [N = 65] is not included in these analyses.) We observed that subjective ratings of pain intensity did not vary significantly between the five scenario prompts (*F*(4,189) = 0.34, *p* = .848, η_p_^2^ = .01). Moreover, the prompts did not influence pain specificity (*F*(4,189) = 1.66, *p* = .162, η_p_^2^ = .03), or subjective ratings of how believable (*F*(4,189) = 1.35, *p* = .252, η_p_^2^ = .03) or how posed expressions looked (*F*(4,189) = 2.13, *p* = .079, η_p_^2^ = .04).

**Correspondence between self-reported race and perceived race.** We note that models’ self-reported race did not always match up with their modal categorization. Our workbook of norming data provides information on whether these measures matched or not. Not surprisingly, targets whose self-reported race did not match their modal categorization were also rated as looking less racially prototypic (*F*(1,262) = 61.33, *p* < .001, η_p_^2^ = .19; *M*_NoMatch_ = 3.53, *SD*_NoMatch_ = 0.40, N = 48), compared to targets for whom these measures matched (*M*_Match_ = 4.01, *SD*_Match_ = 0.38, N = 216).

**Correspondence between coded expression type and objective measurements related to eye and mouth regions.** Beyond the high correspondence between coders’ judgments of expression type, we note a strong relationship between these judgments and objective measurements of mouth and eye width-to-height ratios in emotional expressions. Specifically, mWHR varied as a function of expression type (*F*(3,656) = 31.73, *p* < .001, η_p_^2^ = .13), with the largest values observed for eyes-closed-mouth-closed expressions (*M* = 6.18, *SD* = 8.56; all comparisons to subsequent cells *p* < .005), then for eyes-open-mouth-closed expressions (*M* = 4.55, *SD* = 3.71; all comparisons to subsequent cells *p* < .001), followed by eyes-closed-mouth-open (*M* = 2.11, *SD* = 0.70) and eyes-open-mouth-open expressions (*M* = 1.98, *SD* = 0.59). Both leWHR (*F*(3,656) = 231.55, *p* < .001, η_p_^2^ = .51) and reWHR (*F*(3,656) = 272.75, *p* < .001, η_p_^2^ = .56) also varied as a function of expression type, with the largest values observed for eyes-closed-mouth-closed expressions (*M_LE_* = 19.57, *SD_LE_* = 9.57; *M_RE_* = 21.91, *SD_RE_* = 9.61; all comparisons to subsequent cells *p* < .001), then for eyes-closed-mouth-open expressions (*M_LE_* = 17.12, *SD_LE_* = 8.51; *M_RE_* = 19.35, *SD_RE_* = 9.19; all comparisons to subsequent cells *p* < .001), followed by eyes-open-mouth-closed (*M_LE_* = 5.32, *SD_LE_* = 2.60; *M_RE_* = 5.57, *SD_RE_* = 3.38) and eyes-open-mouth-open (*M_LE_* = 4.81, *SD_LE_* = 2.31; *M_RE_* = 5.11, *SD_RE_* = 3.06) expressions.

**Additional FACS coding results across the full DPD**. The five reliable and pain-relevant AUs detailed above were still significantly more likely to be present in the full set of pain-categorized images versus the full set of non-pain-categorized images (AU04: pain-categorized = 86.8%, non-pain = 58.4%; AU06: pain = 82.4%, non-pain = 59.8%; AU07: pain = 90.8%, non-pain = 72.6%; AU09: pain = 71.3%, non-pain = 41.8%, AU45: pain = 81.3%, non-pain = 51.6%; all *p*s < .00001). Moreover, the pain index we calculated across these five AUs was still strongly positively correlated with our naïve raters’ judgments of pain intensity (*r* = .484, *p* < .0001) across the full sample of expressions.

**Assessing in-group bias in pain ratings.** Overall, correspondence between perceivers’ sets of ratings was high across rater race. White perceivers’ ratings were positively correlated across the full set (of White, Black, Asian, and Hispanic) expressions with Black raters (*r* = .715), Asian raters (*r* = .720), and Hispanic raters (*r* = .624). Black raters’ ratings were correlated with Asian (*r* = .577) and Hispanic raters’ ratings (*r* = .499), and Asian raters’ ratings were correlated with Hispanic raters’ ratings (*r* = .504; all *p*s < .0001). These relationships stayed robust and positive when assessed within each set of models (e.g., correlations ranged from *r* = .345 [between Asian and Hispanic raters’ ratings of Asian models; *p* < .0001] to *r* = .779, [between Black and White raters’ ratings of White models; *p* < .0001]).

Next, we conducted a mixed factorial ANOVA assessing the effects of model race (a between-targets factor) and rater race (a within-targets factor) on ratings of pain intensity, specifically within pain-categorized expressions. Neither the main effects of model race (*F*(3,723) = 1.27, *p* = .282, η_p_^2^ = .005) nor the main effect of perceiver race (*F*(3,241) = 0.78, *p* = .506, η_p_^2^ = .010) was statistically significant. Critically, the interaction between model race and rater race (*F*(9,723) = 1.14, *p* = .335, η_p_^2^ = .014) was also not significant.

Moving forward, we explicitly assessed whether there were in-group biases in pain ratings within each group of raters. Despite the non-significant interaction between model and rater race, general patterns in the data suggested that raters were rating pain expressions from targets of their own race higher in intensity than targets from other races (White raters: *M*_WhitePain_ = 4.27 [*SD* = 0.92], *M*_NonWhitePain_ = 4.04 [SD = 0.76], *p* = .030; *M*_BlackPain_ = 4.33 [*SD* = 1.11], *M*_NonBlackPain_ = 4.14 [*SD* = 1.27], *p* = .277; *M*_HispanicPain_ = 4.15 [*SD* = 1.63], *M*_NonHispanicPain_ = 3.91 [*SD* = 1.52], *p* = .430; *M*_AsianPain_ = 4.00 [*SD* = 1.31], *M*_NonAsianPain_ = 4.04 [*SD* = 1.33], *p* = .886). That said, this effect was only significant within White targets.

Taken together, these data suggest that raters belonging to different racial groups show similar patterns of ratings of pain intensity, both across all models *and* when split by model race. While there was no systematic interaction between rater race and model race on pain intensity ratings within pain-categorized images, when we simplified this analysis to compare ratings of in-group versus out-group models, some evidence of in-group bias emerged within White raters. However, as we note in the main text, if pain is being slightly underestimated on non-White models’ faces, then any comparison between White and non-White models equated on pain ratings would actually make for a *more* conservative test of racial bias in pain perception.

**Study 2: Supplementary Materials**

**Additional information regarding expression creation**

Our initial approach was to create a wide variety of 41 expressions that could potentially be perceived as representing pain using FaceGen Modeller v3.5 (Singular Inversions, https://facegen.com/modeller.htm), to collect norming data characterizing subjective perceptions of these expressions, and then to select a subset of these expressions (which were maximally and specifically painful) for use in subsequent investigations. To accomplish this goal, we used real photographs of the actors of the DPD as our guides and manipulated sliders corresponding to emotional expressions (e.g., anger, disgust), dynamic changes in facial features (e.g., eye squint, brow raise), and vocal production of phonemes (e.g., “ooh”, “aah”). For comparison, we also created a series of decoy expressions representing other emotions (e.g., happiness, sadness, etc.) to be intermixed and rated with our novel painful expression. Expressions were presented on the average face in FaceGen and exported in .png format.

**Additional information regarding stimulus norming**

**Subsequent norming of FaceGen Modeller Pro expressions.** 78 participants (38 female, mean age = 33.16, *SD* = 9.38; 53 White/Caucasian, 11 African American, 8 Asian, 5 Hispanic, 1 Native American) were recruited from Mechanical Turk and given the task of rating a random sampling of 30 expressions out of 5 angry expressions, 10 fearful expressions, 5 happy expressions, 12 sad expressions and 26 painful expressions (the 11 expressions detailed in main text—now recreated in FaceGen Modeller Pro—as well as 15 other painful expressions of lower intensity). Each expression received an average of 40.34 ratings per face. Expression order was randomized; dimension order was randomized within expression.

We note two changes in the procedure of this norming task. First, in this second norming task, we made a small change to the wording of pain rating item. In the first norming survey, participants responded to the question “How much does this face look like it’s in physical pain?”, which differed in its construction from other emotions (e.g., “How fearful does this face look?”). For parallel construction, we changed the pain wording to “How physically in pain does this face look?” This change did not significantly impact the overall pattern or intensity of ratings of painful expressions. Moreover, in this second norming, we chose to collect ratings of confusion instead of threat.

**Subsequent norming of FaceGen Modeller Pro expressions on an average Black face.** In order to confirm that the expressions of pain we created in FaceGen are, indeed, seen as pain on Black faces, we have conducted a new, separate norming of our computer-generated stimuli. We recreated the 11 selected pain expressions we originally normed, along with 15 additional pain expression variants and 8 other emotional expressions (2 angry, 2 sad, 2 fear, and 1 happy expression) and rendered them in FaceGen Modeller Pro on an average Black face. 45 paid Prolific participants (23 female, *M*_age_=32.13, *SD*=14.55, 23 White/Caucasian, 6 African American, 4 Asian, 7 Hispanic, 1 Native American, 4 identifying otherwise) rated these 33 stimuli in a randomized order. As we did in our original procedure, we calculated average ratings for each emotion (anger, fear, disgust, sadness, happiness, surprise, confusion, and physical pain) for each expression.

**Additional results**

**FaceGen Modeller Pro norming.** Here we offer expanded results characterizing comparisons of pain ratings to other emotion ratings for the eleven painful expressions detailed in the main text.

Each of these eleven painful expressions was rated above the scale midpoint in terms of painfulness (all *M*s > 4.65), and was rated as resembling pain more so than anger (all *p*s < .0031; all *Ms* < 4.38), disgust (all *p*s < .0002; all *Ms* < 3.18), fear (all *p*s < .0001; all *Ms* < 2.90), happiness (all *p*s < .0001; all *Ms* < 2.07), sadness (all *p*s < .0002; all *Ms* < 3.13), surprise (all *p*s < .0001; all *Ms* < 2.38), or threat (all *p*s < .0001; all *Ms* < 3.35). Averaged ratings across the set suggested that these eleven expressions were clearly rated as resembling pain (*M* = 5.18, *SD* = 0.36), rather than any other emotion (anger: *M* = 2.41, *SD* = 0.75; disgust: *M* = 2.69, *SD* = 0.30; fear: *M* = 2.25, *SD* = 0.30; happiness: *M* = 1.48, *SD* = 0.33; sadness: *M* = 2.24, *SD* = 0.42; surprise: *M* = 1.89, *SD* = 0.28; threat: *M* = 2.04, *SD* = 0.51).

Notably, multiple versions of FaceGen exist, and the implementation of the sliders used to create these expressions varies across versions, including the premium version—FaceGen Modeller Pro. However, the Pro version offers one important advantage, in that researchers can simply save expressions, rather than recreating them by hand using the sliders described above. As an additional step, we attempted to recreate these eleven painful expressions using the Pro version, and subsequently subjected them to a second norming survey (for sample details, see above).

Once more, each expression was rated above the scale midpoint in terms of painfulness (average *M =* 4.85, all *M*s > 4.13), and was rated as resembling pain more so than anger (average *M =* 2.72, all *Ms* < 3.00, all *p*s < .002), disgust (average *M =* 3.00, all *Ms* < 3.41, all *p*s < .004), fear (average *M =* 2.62, all *Ms* < 3.38, all *p*s but fear < .001; *p*_fear_ = .072), happiness (average *M =* 1.97, all *Ms* < 2.56, all *p*s < .0001), sadness (average *M =* 2.56, all *Ms* < 3.17, all *p*s < .005), surprise (average *M =* 2.36, all *Ms* < 3.00, all *p*s < .011), or confusion (average *M =* 2.39, all *Ms* < 3.13, all *p*s < .012).

**FaceGen Modeller Pro norming on an average Black face.** We first assessed the degree of correlation between pain intensity ratings for all 33 expressions included in our final norming survey (e.g., where expressions were rendered on an average Black face in FaceGen Modeller Pro) with ratings of the same expressions in the previous survey. This relationship was very strongly positive (*r* = .929, *p* < .0001). Within the 11 selected pain expressions described above, this correlation remained strong and positive (*r* = .597, *p* = .052).

Next, we assessed correspondence between how the two sets of expressions were judged. We categorized each expression in terms of which emotion it received the highest intensity ratings on in either norming surveys—e.g., an expression that got an average rating of 4 for physical pain and an average rating of 2 for all other emotions would be categorized as physical pain. In 25 of 33 cases, there was agreement across the two sets of ratings (e.g., those collected from the average face and those collected from the average Black face). Moreover, in the 8 cases where there *was* divergence, only two of these were originally seen as pain on the average face and not on the average Black face, and further, only one of these was one of our selected 11 expressions. (We discuss this expression below.)

Within the 11 selected pain expressions, the patterns of means across the eight rated emotions were extremely highly correlated with each other across the two norming sets (*r* = .952, *p* < .0001). Moreover, within the norming set using the average Black face, these expressions were, on average, rated as resembling pain more than any other emotion (*M*_pain_ = 4.32; *M*_fear_ = 2.38, *M*_anger_ = 2.63, *M*_disgust_= 3.14, *M*_happiness_= 1.38, *M*_sadness_ = 2.61, *M*_surprise_ = 1.89, *M*_confusion_ = 2.24; all *p*s < .0031 [anger]).

On average, means for ratings of physical pain *were* lower when expressions were rated on the average Black face versus the average face (*M*_pain_averageface_ = 4.85, *M*_pain_averageBlackface_ = 4.32; *p* = .003). Indeed, in one case, one expression (“AK5”) was still rated significantly higher on pain than any other emotion when rendered on the average Black face, but its average physical pain rating was below the scale’s midpoint (*M*_pain_ = 3.44). That said, this is perfectly consistent with our own work suggesting that pain is less readily perceived on Black versus White faces [6]. A greater concern would arise if any of these expressions of pain were not seen as representative of pain when rendered on Black faces, but rather, were seen robustly as some other emotion. Only one expression (“PMS13”) fit this pattern. PMS13 was rated significantly higher on anger than pain (*M*_pain_ = 3.31, *M*_anger_ = 5.78, *p* < .0001). We recommend that researchers wishing to conduct a more conservative test of race-based differences in pain perception avoid using this particular expression. As such, we have flagged this expression in all posted online materials relevant to the stimuli in Study 2.

**References**

[1] Baltrušaitis, T., Mahmoud, M., & Robinson, P. Cross-dataset learning and person-specific normalisation for automatic action unit detection. In 2015 11th IEEE International Conference and Workshops on Automatic Face and Gesture Recognition (FG; 2015 May) (Vol. 6, pp. 1-6). IEEE.

[2] Baltrusaitis T, Zadeh A., Lim YC, Morency, LP. Openface 2.0: Facial behavior analysis toolkit. In 2018 13th IEEE International Conference on Automatic Face & Gesture Recognition (FG; 2018 May) (pp. 59-66). IEEE.

[3] Chen C, Crivelli C, Garrod OG, Schyns PG, Fernández-Dols JM, Jack RE. Distinct facial expressions represent pain and pleasure across cultures. Proc Natl Acad Sci U S A. 2018 Oct 23;115(43):E10013-21.

[4] Ekman P, Rosenberg, E. (1997). What the face reveals: Basic and applied studies of spontaneous expression using the Facial Action Coding System (FACS). Oxford University Press, USA. 1997.

[5] Kunz M, Meixner D, Lautenbacher, S. Facial muscle movements encoding pain—a systematic review. Pain. 2019 Mar;160(3):535-549.

[6] Mende-Siedlecki P, Qu-Lee J, Backer R, Van Bavel JJ. Perceptual contributions to racial bias in pain recognition. J Exp Psychol Gen. 2019 May;148(5):863-889.

[7] Prkachin KM, Solomon, PE. The structure, reliability and validity of pain expression: Evidence from patients with shoulder pain. Pain. 2008 October 15;139(2):267-274.

1. As we note above, the vast majority (>98%) of rated expressions were posed at a level 8 – though there was considerable variability in the perceived intensity of those expressions and the extent to which they were recognized as conveying pain. As a result, very few 5 out of 10 expressions were rated (N = 13). That being said, it would be concerning if the description of the 5 out of 10 prompt (“you can grit your teeth through it”) primed a certain type of expression that was ultimately overrepresented in our set. To address this, we compared the number of Mouth Open vs. Mouth Closed expressions that were ultimately rated. Across the full set of expressions that were rated, 52.6% were posed with mouths open (which would include visibly gritted teeth) and 47.4% were posed with mouths closed. Within pain-categorized expressions, 50.4% were posed with mouths open and 49.4% were posed with mouths closed. Taken together, it seems unlikely that the wording of the 5 out of 10 level description unduly affected the types of expressions that models posed. [↑](#footnote-ref-1)
